# *Haloferax mediterranei*, an Archaeal Model for Denitrification in Saline Systems, Characterized Through Integrated Physiological and Transcriptional Analyses

**DOI:** 10.3389/fmicb.2020.00768

**Published:** 2020-04-22

**Authors:** Javier Torregrosa-Crespo, Carmen Pire, Linda Bergaust, Rosa María Martínez-Espinosa

**Affiliations:** ^1^Department of Agrochemistry and Biochemistry, Faculty of Science, University of Alicante, Alicante, Spain; ^2^Faculty of Chemistry, Biotechnology and Food Science, Norwegian University of Life Sciences, Ås, Norway

**Keywords:** *Haloferax*, denitrification, nitrogenous gases, nitrate reductase, nitrite reductase, extremophile

## Abstract

*Haloferax mediterranei* (R4) belongs to the group of halophilic archaea, one of the predominant microbial populations in hypersaline environments. In these ecosystems, the low availability of oxygen pushes the microbial inhabitants toward anaerobic pathways and the presence of N-oxyanions favor denitrification. In a recent study comparing three *Haloferax* species carrying dissimilatory N-oxide reductases, *H. mediterranei* showed promise as a future model for archaeal denitrification. This work further explores the respiratory physiology of this haloarchaeon when challenged with ranges of nitrite and nitrate concentrations and at neutral or sub-neutral pH during the transition to anoxia. Moreover, to begin to understand the transcriptional regulation of N-oxide reductases, detailed gas kinetics was combined with gene expression analyses at high resolution. The results show that *H. mediterranei* has an expression pattern similar to that observed in the bacterial Domain, well-coordinated at low concentrations of N-oxyanions. However, it could only sustain a few generations of exponential anaerobic growth, apparently requiring micro-oxic conditions for *de novo* synthesis of denitrification enzymes. This is the first integrated study within this field of knowledge in haloarchaea and Archaea in general, and it sheds lights on denitrification in salty environments.

## Introduction

The ability to maintain a respiratory metabolism in lieu of oxygen is widespread. Among the many types of anaerobic dissimilatory pathways, denitrification is the most energetically profitable. Complete denitrification is the stepwise reduction of nitrate (NO_3_^–^) to dinitrogen (N_2_) via nitrite (NO_2_^–^), nitric oxide (NO), and nitrous oxide (N_2_O) (Zumft and Kroneck, 2006; Philippot et al., 2007; Bakken et al., 2012). Despite its ubiquity, our knowledge about the “nuts and bolts” of the process is based on detailed studies of a few model organisms, mainly proteobacteria with mesophilic lifestyles. The role of denitrification in nitrogen turnover and N-oxide emission have been studied at length in environments such as agricultural or forestry soils (Bru et al., 2007; Samad et al., 2016; Roco et al., 2017). Less is known about saline and hypersaline ecosystems, where anaerobic respiration is prominent because the high salt concentrations result in low oxygen availability (Rodríguez-Valera et al., 1985; Sherwood et al., 1991, 1992; Oren, 2013). Such systems are becoming increasingly interesting in terms of denitrification and N-oxide emissions because anthropogenic activities currently lead to contamination by nitrogenous compounds like nitrates and nitrites (Martínez-Espinosa et al., 2007; Ochoa-Hueso et al., 2014; Torregrosa-Crespo et al., 2018). Moreover, their extent on a global scale is on the rise due to desertification, resulting from climate change (Torregrosa-Crespo et al., 2018).

Hypersaline environments accommodate representatives of all the three Domains of Life, but when the salt concentration exceeds 16%, haloarchaea dominate (Andrei et al., 2012; Edbeib et al., 2016; Torregrosa-Crespo et al., 2019). Among them are several denitrifiers and the microorganism that has emerged as a representative model is *Haloferax mediterranei*. It has the full set of metalloenzymes to catalyze the reduction of NO_3_^–^ to N_2_: the membrane-bound nitrate reductase facing the pseudo periplasm (NAR) (Lledó et al., 2004; Martínez-Espinosa et al., 2007), the copper-containing nitrite reductase (NIR) (Martínez-Espinosa et al., 2006), the nitric oxide reductase (NOR) (Torregrosa-Crespo et al., 2017), and the nitrous oxide reductase (N_2_OR) (Torregrosa-Crespo et al., 2016). Currently, it is one out of very few archaea for which detailed phenotypic data exist, and it is the only one which has been shown to be a complete denitrifier, able to reduce NO_3_^–^ to N_2_ while displaying low and transient accumulation of the gaseous intermediates NO and N_2_O (Torregrosa-Crespo et al., 2019). This opens the door to further exploration of its respiratory physiology and regulation of denitrification.

Like its bacterial counterparts, *H. mediterranei* carry *nar*, *nir*, *nor*, and *nos* gene clusters encoding the four structural enzymes of denitrification (Lledó et al., 2004; Martínez-Espinosa et al., 2006; Torregrosa-Crespo et al., 2016). However, unlike some of the well-studied proteobacteria (Bergaust et al., 2008; Qu et al., 2016), little is known about the expression of the functional denitrification genes during the transition from aerobic to anaerobic respiration, or the effect of pH and electron acceptor concentrations on N-oxide accumulation. Through a combination of gene expression analyses and detailed physiological studies, this work begins to remedy this, finding evidence to suggest that regulatory similarities exist across domains. Thus, it bears implications for our understanding of denitrification in archaea in general, and in a representative of the halophilic community in particular.

## Materials and Methods

### Physiological Experiments Testing Different NO_3_^–^ and NO_2_^–^ Concentrations and pH Values

#### Culturing Conditions

*Haloferax mediterranei* (R4) was raised in a complex medium [20% (w/v) mixture of salts and 0.5% (w/v) yeast extract] (Rodríguez-Valera et al., 1980; DasSarma et al., 1995), pH 7.3 at 35°C under aerobic conditions, with vigorous stirring (700 r/min) using a triangular magnetic stirring bar (Cowie 25 × 8 mm, VWR International) to ensure full dispersal of cells and oxic conditions. Moreover, the aerobic pre-cultures were never allowed to grow beyond densities of 1.94 × 10^8^ cells mL^–1^.

Aerobic pre-cultures were transferred to 120 ml serum vials containing a triangular magnetic stirring bar and 50 ml medium at 35°C. All cultures were continuously stirred at 700 r/min, to avoid aggregation. The media contained different KNO_3_ or KNO_2_ concentrations depending on the assay: 2, 5, 10, 20, 200, and 2000 mM KNO_3_; 1, 2, 5, 10, and 40 mM KNO_2_. The pH was 7.3 (optimum for *H. mediterranei*) for all the experiments and the media were always buffered with Bis-Tris 100 mM. The different pH values for the experiments to test the effect of this parameter on denitrification were: 7.3, 7.0, 6.5.

Vials were crimp sealed with rubber septa (Matriks AS, Norway) and aluminum caps to ensure an airtight system. The vials were made anoxic by repeated cycles of evacuation and helium (He) filling with constant stirring to ensure optimal gas exchange between liquid and headspace. The initial availability of gaseous electron acceptors was then adjusted by injection of pure O_2_ and in some instances N_2_O. All experiments were conducted under atmospheric pressure at 35°C.

#### Gas Measurements and Apparent Specific Growth Rates

The gas measurements were done in a robotized incubation system similar to that described by Molstad et al. (2007), with some improvements. The system monitored the headspace concentrations of relevant gases (O_2_, CO_2_, NO, N_2_O, and N_2_) by repeated gas sampling through the butyl rubber septa of the incubation vials (30 stirred vials). The gas samples were drawn by a peristaltic pump coupled to an autosampler (Agilent GC Sampler 80), and with each sampling an equal volume of He was pumped back into the vials. This secured that the gas pressure was sustained near 1 atm despite repeated sampling but diluted the headspace atmosphere (with He). This dilution was considered when calculating the rates of production/consumption for each time increment (see Molstad et al., 2007 for details). The sampling system was coupled to a gas chromatograph (GC) (Agilent GC–7890A) with 30 m 0.53 mm id columns: a porous layer open tubular (PLOT) column for separation of CH_4_. NO was measured by a chemiluminescence NO_x_ analyzer (M200A or M200E, Teledyne).

Nitrite was measured by injecting 10 μl liquid sample into a purge vessel containing 3 ml reducing agent (NaI, 1% w/v in acetic acid) connected to a chemiluminescence detector (Nitric Oxide Analyzer NOA 280i, General Electric). N_2_ was continuously bubbled through the reducing agent to maintain an anoxic environment in the system and to transport the NO through the NO analyzer (Walters et al., 1987).

Apparent specific aerobic (μ_ox_) and anaerobic (μ_*an*__ox_) growth rates were estimated based on the observed kinetics of O_2_ consumption and denitrification, respectively, through the regression of log (ln) transformed respiration rates versus time during log-linear increases in electron transport rates.

### Gas Kinetics and Transcription of *narG*, *nirK*, *norZ*, and *nosZ* Genes During O_2_ Depletion and Subsequent Denitrification

A total of 15 anoxic vials containing media supplemented with 5 mM KNO_3_ were injected with pure O_2_ to approximately 1 vol% in headspace and then inoculated with 2 ⋅ 10^8^ cells from aerobic pre-cultures. Cultures were placed in the robotized incubation system at 35°C, with continuous stirring at 700 r/min, and the gas kinetics in each vial was monitored by headspace measurements (1- to 3-h intervals). Samples for gene expression analysis (50, 10, 5, or 2 ml depending on cell density) were harvested at frequencies guided by the observed gas kinetics; at each time, cells were taken from three separate vials and considered biological replicates. Entire vials were sacrificed for 50 and 10 ml samples, whereas a maximum of two 5 ml samples were taken before vials were excluded from further gas measurements. Nitrite concentration and cell densities (OD_600_) were measured for each sample. Three replicates were left undisturbed by liquid sampling and only headspace gases were monitored.

#### Gene Expression Analysis

Guided by the observed gas kinetics, samples (50, 10, 5, or 2 ml, depending on cell density) were taken from the liquid phase of the vials throughout the experiment, as well as from the inoculum (time: 0 h). Samples were transferred to chilled, sterile 50 or 2 ml centrifugation tubes. One milliliter of the sample was taken to measure OD_600_ and NO_2_^–^ concentration. The remaining sample volume was pelleted by centrifugation (10,000 × *g*, 10 min) at 4°C. The supernatant was decanted, and 1 ml of a mixture of RNA protect Bacteria Reagent (Qiagen) with salty water 20% w/v (Rodríguez-Valera et al., 1980; DasSarma et al., 1995) in a 1:1 ratio was added to the cell pellet. After treatment with RNAprotect, the cells were pelleted once more by centrifugation (10,000 × *g*, 10 min) at room temperature and then stored at −20°C awaiting RNA extraction. Due to the considerable number of samples, the biological replicates were divided into three times series; RNA extraction and DNAse treatment were done in three rounds, whereas for reverse transcription, all samples were collected and processed in one round. Total RNA was extracted from all samples using the RNeasy Mini Kit (Qiagen) and residual DNA removed by DNase treatment (TURBO DNA-free, Ambion) according to the suppliers’ instructions. The concentration of total RNA was measured using the Qubit RNA BR assay and a Qubit fluorometer (Invitrogen). Absence of gDNA was confirmed in each sample by real-time PCR targeting the *norZ* gene, using RNA that had not been reverse transcribed. Reverse transcription was performed using a SuperScript VILO cDNA Synthesis Kit (Invitrogen). Droplet Digital PCR (ddPCR)^TM^ was performed on a QX200 ddPCR^TM^ System (Bio-Rad) using Bio-Rad’s QX200 ddPCR EvaGreen supermix and primers to target *narG*, *nirk*, *norB*, and *nosZ* (Supplementary Table S3). The expression of each gene was calculated by absolute quantification. ddPCRs were set up in technical triplicate for each of the three biological replicates and normalized by (RNAtotal) ng sample^–1^.

### Aerobic N_2_O Reduction in Early Denitrification

Pre-culturing and gas measurements of batch cultures were carried out as described in Section “Physiological Experiments Testing Different NO_3_*^–^* and NO_2_*^–^* Concentrations and pH Values.” The aerobically raised cells were transferred to He-rinsed vials containing 50 ml complex medium with 5 mM KNO_3_, and with 1 vol% O_2_ 100 ppmv N_2_O (in triplicate). Cell-free vials were also included in triplicate for each treatment to provide accurate estimates of sampling dilution. Gas kinetics was monitored every 3.6 h in the robotized incubation system described under Section “Gas Measurements and Apparent Specific Growth Rates.”

## Results

### Effect of Nitrate Concentration on Denitrification in *H. mediterranei*

**FIGURE 1:**
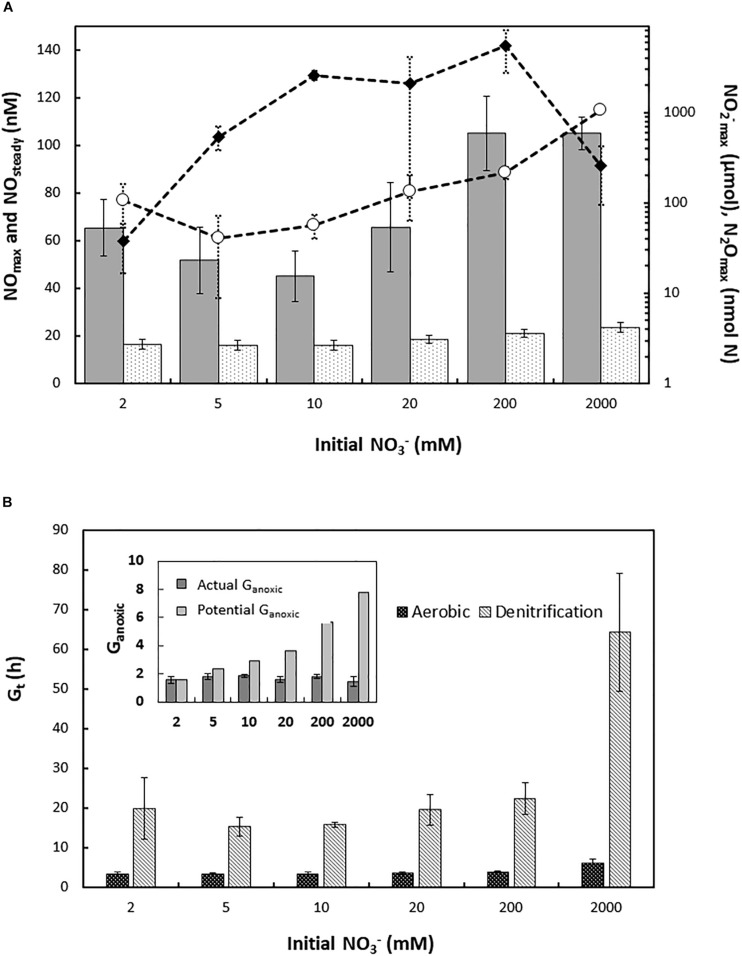
**(A)** Accumulation of N-oxide intermediates during denitrification in batch cultures of *H. mediterranei* supplemented with different initial KNO_3_ concentrations (2–2000 mM). Bars, left *y*-axis: NO_max_ (gray bars) and NO_steady_ (dotted bars; average NO concentration during exponential anaerobic growth), nM in liquid; plots, right *y*-axis (log-scale): NO_2_^–^ (black diamonds) and N_2_O_max_ (circles), μmol and nmol N vial^–1^, respectively. **(B)** Generation time (G_t_, h) and duration of apparent exponential growth by denitrification in *H. mediterranei* supplemented with 2–2000 mM KNO_3_. Main panel: generation time (h) during aerobic respiration (black bars) and denitrification (gray patterned bars), derived from apparent specific growth rates (μ h^–1^). Oxic and anoxic μ h^–1^ were estimated based on the ln transformed e-flow rate to terminal electron acceptors during the exponential phase of oxygen respiration and denitrification, respectively. Insert: estimated actual (dark gray bars) and potential (light gray bars) generations of exponential growth by denitrification (G_anoxic_) across nitrate treatments. For the cultures with 2 M nitrate, denitrification rate was still exponential at the end of the experiment, leading to a likely underestimation of actual G_anoxic_.

*Haloferax mediterranei* was exposed to different initial concentrations of nitrate (ranging from 2 mM to 2 M KNO_3_) with 1 vol% initial O_2_ in the headspace, and aerobic respiration and denitrification was monitored by frequent gas measurements (Molstad et al., 2007). Nitrate concentrations did not severely affect the accumulation of gaseous intermediates during the oxic-anoxic transition or subsequent denitrification (Figure 1A and Supplementary Table S1A). However, the two most extreme treatments (0.2 and 2 M initial nitrate) led to a subtle increase in the transient NO peak following oxygen depletion (NO_max_) as well as in the average NO concentration during balanced anaerobic growth (NO_steady_). There was an approximate doubling and ten-fold increase in the accumulation of N_2_O in 0.2 and 2 M cultures, respectively, compared to 2–20 mM cultures, with observed N_2_O_max_ of 0.21 ± 0.03 and 1.06 ± 0.08 μmol N_2_O-N vial^–1^ in the high nitrate cultures (Supplementary Table S1A). The accumulation of nitrite (NO_2_^–^_max_) increased with initial nitrate concentration up to 10 mM but was apparently not affected beyond that point. The apparent decrease in cultures with 2 M initial nitrate most likely does not reflect the actual NO_2_^–^_max_, because nitrite was still increasing at the end of the experiment (Figure 1A).

The e- flow toward terminal electron acceptors (Ve^–^, μmol vial^–1^h^–1^) was derived from the gas data and used to estimate the apparent specific growth rates during aerobic respiration and denitrification, μ_ox_ and μ_anox_ h^–1^, respectively. μ_ox_ was largely unaffected by nitrate concentrations up to 0.2 M (μ_ox_ = 0.201 ± 0.020 h^–1^; average generation times G(t) ∼ 3.5 h). However, cultures with 2 M nitrate displayed a clear decrease in μ_ox_ (0.115 ± 0.019 h^–1^) to a near doubling in G(t) (∼6.0 h) (Figure 1B and Supplementary Table S1B). Likewise, μ_anox_ and thus G(t) during denitrification was not significantly affected by nitrate within the range of 2–200 mM (Figure 1B, Supplementary Table S1B, and Supplementary Figure S1), although a weak negative trend was observed (Supplementary Figure S1, insert). However, the cultures with 2 M nitrate showed a dramatic decrease in apparent anaerobic growth rate, with an approximate 300% increase in average generation time relative to 2–200 mM cultures (Figure 1B).

**FIGURE 2:**
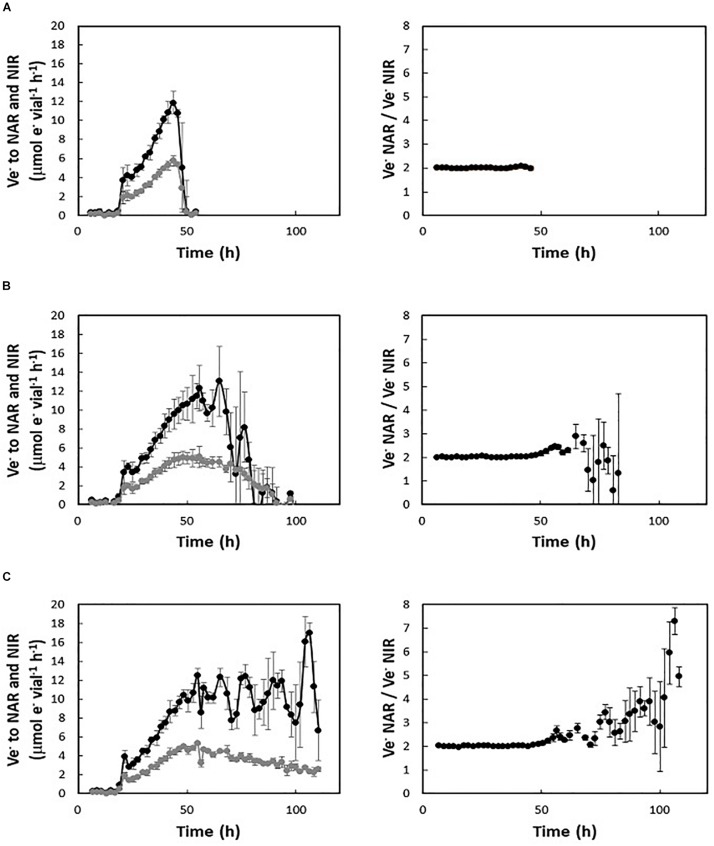
Electron flow (V_e__–_, μmol e^–^ vial^–1^ h^–1^) to respiratory nitrate reductase (NAR—black lines) and nitrite reductase (NIR—gray lines) during the oxic-anoxic transition in batch cultures with 1% initial O_2_ and different initial KNO_3_ concentrations (left panels): 2 mM **(A)**, 5 mM **(B)**, 10 mM **(C)**. Right panels show the respective ratios between the electron flow to NAR and NIR.

The addition of high amounts of nitrate should support multiple generations of exponential anaerobic growth (G_anoxic_), in proportion to initial nitrate concentration. However, regardless of the amount of nitrate available, *H. mediterranei* did not maintain exponential growth by denitrification beyond 1.5–2 generations (Figure 1B, insert). The electron flow (μmol e^–^ vial^–1^ h^–1^) toward NAR and NIR were derived from the gas data for cultures supplemented with 2, 5, and 10 mM KNO_3_, respectively (Figure 2). In cultures with 2 mM nitrate (Figure 2A, left panel), both NAR and NIR rates increased exponentially until they stopped due to depletion of nitrate, whereas in cultures with 5 and 10 mM initial nitrate (Figures 2B,C, left panels), NIR reached a plateau stage first, while the relative NAR activity was still increasing. Toward the end of the incubations in cultures with 5 mM nitrate, NAR activity stopped before NIR due to nitrate depletion. During balanced denitrification where NAR and NIR operate at equal rates, Ve-NAR/Ve-NIR = 2 (two electrons to reduce NO_3_^–^ to NO_2_^–^ and one electron to reduce NO_2_^–^ to NO). In cultures supplemented with 2 mM KNO_3_, the ratio was 2 throughout the incubation (Figure 2A, right panel), whereas in the rest of the cultures, it increased when the electron flow toward NIR reached the plateau (Figures 2B,C, right panels), reflecting higher NAR activity relative to NIR. Injection of O_2_ and NO after decline in denitrification rates had no significant effect, although there was a slight increase in e- flow to N-oxides after addition of O_2_ (Supplementary Figure S2).

The 5 mM nitrate treatment was selected for further exploration of denitrification in *H. mediterranei*: effect of pH on denitrification and transcription of functional denitrification genes during transition to anoxia and subsequent anaerobic growth.

In order to test the effect of pH on denitrification, *H. mediterranei* was exposed to a range of pH levels (7.3, 7.0, 6.5, 6.0, and 5.7) using 100 mM Bis-Tris as buffer. *H. mediterranei* managed the transition to denitrification without any dramatic pH effects at pH ≥ 6.5, but at pH < 6.5, there was a general collapse of respiration upon oxygen depletion and the organism appeared unable to initiate denitrification (Supplementary Table S2A).

### Effect of Nitrite Concentration on Denitrification in *H. mediterranei*

**FIGURE 3:**
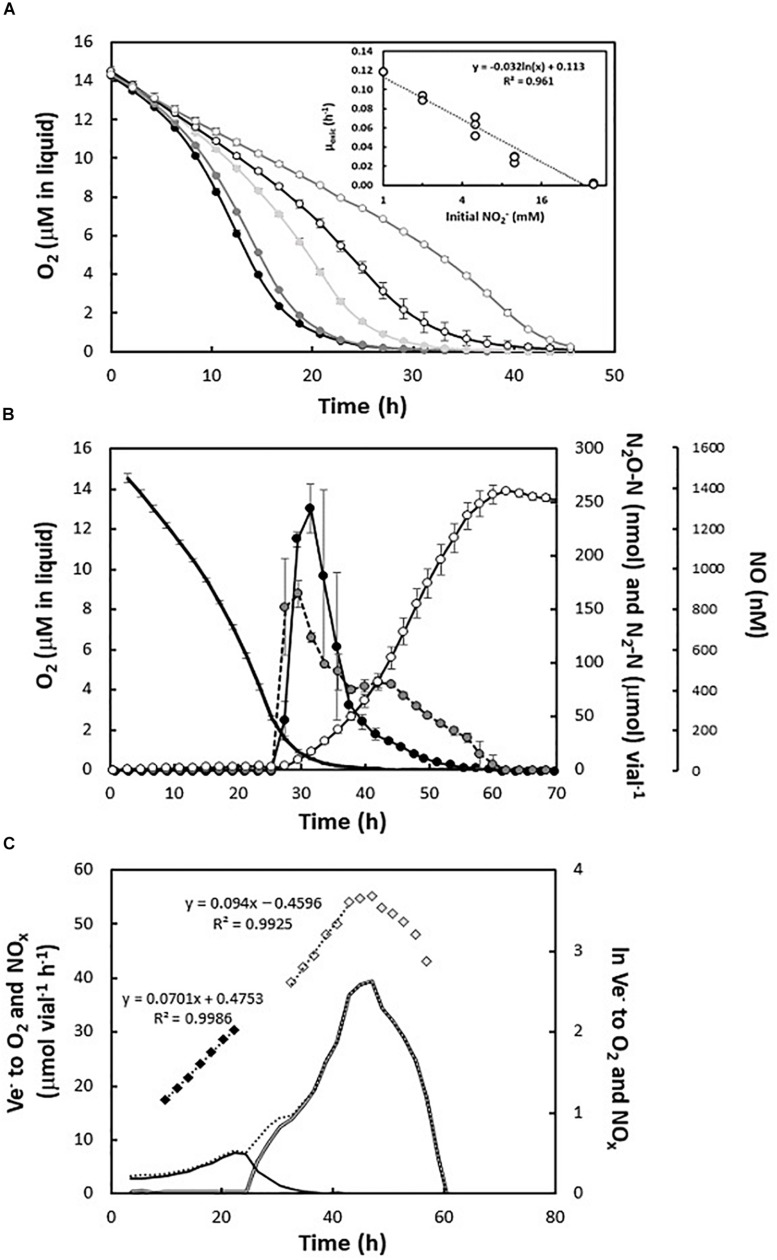
Gas kinetics in cultures supplied with nitrite. **(A)** O_2_ consumption during the incubation of *H. mediterranei* in the presence of 1 mM (black circles), 2 mM (dark gray circles), 5 mM (light gray circles), 10 mM (open black circles), and 40 mM (open gray circles) KNO_2_. Insert: apparent specific anaerobic growth rates (μ_anox_, h^–1^) during log linear growth in the cultures with different initial KNO_2_ concentrations (1–40 mM). **(B)** Detailed gas kinetics during transition to anoxia in nitrite-supplemented medium (5 mM KNO_2_) with 1% initial O_2_ in headspace. Top panel: consumption of O_2_ (black line, no symbols) and subsequent accumulation of N-oxides (NO—closed black circles; N_2_O—closed gray circles; N_2_—open circles). The experiment was conducted at 35°C and in triplicate batch cultures. **(C)** E-flow (V_e–_, μmol vial^–1^ h^–1^) to O_2_ (black line) and N-oxides (double black line) in representative batch culture with 5 mM KNO_2_ and 1% initial O_2_. Diamonds represent the regression of log (ln) transformed respiration rates versus time during log-linear increases in electron transport rates (black diamonds for aerobic respiration and gray diamonds for anaerobic respiration).

A set of experiments was carried out using nitrite (ranging between 1 and 40 mM) as an alternative electron acceptor in the presence of 1 vol% O_2_ in headspace. The presence of nitrite had a negative effect on the O_2_ consumption rate with an apparent log linear decline in μ_ox_ with increasing nitrite concentration (Figure 3A, insert). Thus, while estimated μ_ox_ in cultures with 1 mM initial nitrite was 0.119 ± 0.000 h^–1^ (approximately 50% of the observed rate in nitrite-free cultures), with 10 mM initial nitrite, the specific growth rate declined by approximately 76%, to 0.028 ± 0.004 h^–1^. In cultures with 40 mM initial nitrite, the inhibition was severe with a 98% decrease in apparent aerobic growth rate (0.002 ± 0.001 h^–1^) (Figure 3A).

During the anoxic phase at initial nitrite concentrations between 1 and 5 mM, *H. mediterranei* reduced all the available NO_2_^–^ to N_2_. However, in the presence of 5 mM KNO_2_, the maximum concentration of nitric oxide increased significantly (to approximately 1.4 μM) (Figure 3B). The apparent anoxic growth rate was equal when comparing cultures with 2 and 5 mM of KNO_2_ (μ_anox_ between 0.09 and 0.10 h^–1^ respectively). However, as seen in cultures with ≥ 5 mM nitrate, exponential growth ceased prematurely in the cultures with 5 mM nitrite, while more than half of the added nitrite remained. Likewise, denitrification continued with declining e^–^ flow rates to N-oxides until nitrite depletion (Figures 3B,C and Supplementary Figure S3B). Finally, for the highest concentrations of nitrite tested (10 and 40 mM KNO_2_), *H. mediterranei* accumulated high concentrations of NO (up to 18 μM in the liquid), with subsequent arrest in respiration (Supplementary Figure S4).

Using nitrite as terminal electron acceptor (2 mM), the effect of pH in the slightly acidic to near- neutral range (5.7–7.3) was tested. In this case, low pH resulted in increased maximum concentrations of NO, reaching 490 and 960 nM at pH 7.3 and 6.5, respectively (Supplementary Table S2). At pH 6.5, some cultures were unable to respire nitrite after O_2_ depletion, whereas at pH < 6.5 both aerobic and anaerobic respiration failed.

### Transcription of *narG*, *nirK*, *norZ*, and *nosZ* Genes During O_2_ Depletion and Subsequent Denitrification

The transcription of *narG*, *nirK*, *norZ*, and *nosZ* was monitored alongside gas kinetics and NO_2_^–^ accumulation during the transition from aerobic to anaerobic respiration, and the conspicuous decline in growth rate during late anoxia. The cultures were supplemented with 5 mM initial KNO_3_ and 1% initial O_2_.

**FIGURE 4:**
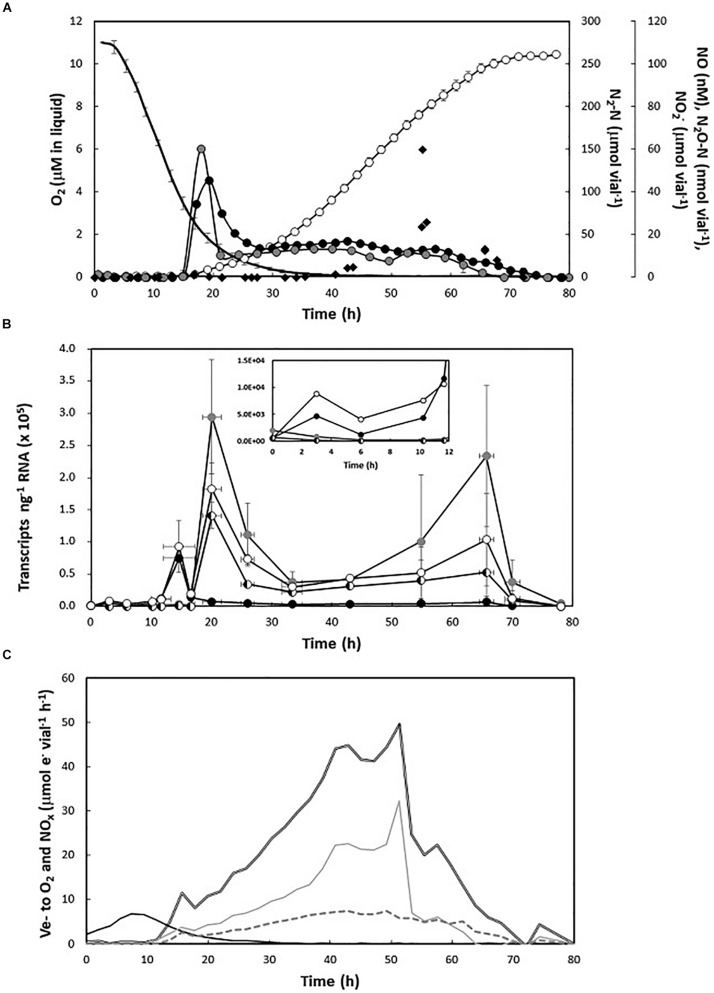
Gas kinetics, gene transcription, and electron flow through the transition from aerobic to anaerobic respiration. **(A)** Consumption of O_2_ (black line, no symbols) and subsequent accumulation of N-oxides (NO_2_^–^—black diamonds; NO—closed black circles; N_2_O—closed gray circles; N_2_—open circles) in nitrate-supplemented medium (5 mM KNO_3_) with 1% initial O_2_ in headspace (*n* = *3*). **(B)** Number of gene transcripts per nanogram RNA which is shown with standard deviation as vertical lines (*n* = *3*) (*narG*—closed black circles; *nirK*—closed gray circles; *norZ*—half white and half black circles; and *nosZ*—open white circles). The large deviations were due to an overall higher signal during active denitrification from one of the three replicate series. The sample at time 0 is the result for the inoculum (prior to inoculation). **(C)** Electron flow (μmol vial^–1^h^–1^) to O_2_ (black line), nitrate reductase (gray line), nitrite reductase (discontinuous gray line), and total N-oxides (double black line).

The observed gas kinetics were as expected for *H. mediterranei* under the relevant conditions (Torregrosa-Crespo et al., 2019). The cultures reduced all available nitrate to N_2_ with minimal and transient accumulation of intermediates. Denitrification was initiated at low O_2_ (2.49 ± 0.28 μM in liquid), as seen by transient peaks of NO and N_2_O (NO_max_: 51.02 ± 0.63 nM; N_2_O_max_: 95.13 ± 5.97 nM), which dropped to background semi-steady state levels that were maintained until depletion of N-oxides. As seen previously, N_2_ increased exponentially during early anoxia, then at linear or declining rate until depletion of added nitrate (Figure 4A). The activity of NirK (seen as e- flow to nitrite, μmol vial^–1^ h^–1^), slowed prior to, and more dramatically than Nar (Figure 4C), which led to transient nitrite accumulation (NO_2_^–^_max_: 67.56 μM vial^–1^) during late anoxia (Figure 4A).

The structural denitrification genes showed contrasting expression profiles. Unlike *nirK* and *norZ* whose expression showed a slight decline during the first 10 h of the incubation, *narG* and *nosZ* transcription both increased by an order of magnitude within the first 3 h after inoculation and remained elevated throughout the semi-aerobic phase (Figure 4B, insert). Immediately before the transient NO and N_2_O peaks, *narG* and *nosZ* transcription increased by another order of magnitude, peaking at 7.5E4 ± 7.3E2 and 9.2E4 ± 4.0E4 copies ng^–1^ RNA, respectively. Transcription of *narG* subsequently decreased and was kept at a reasonably constant number (4.9E3 ± 1.7E3 copies ng^–1^ RNA) during the anoxic phase. As NO peaked, *nosZ* reached a maximum of 1.8E5 ± 4.1E4 copies ng^–1^ RNA. This coincided with high expression of *nirK* and *norZ*, which both increased from background levels (fluctuating around 1E2 copies ng^–1^ RNA) to 2.9E5 ± 8.9E4 and 1.4E5 ± 2.0E4 copies ng^–1^ RNA, respectively. The *nirK*, *norZ*, and *nosZ* transcription maxima were followed by a period of exponential N_2_ accumulation, during which the expression of all three genes gradually dropped by an order of magnitude. As the apparent growth rate by denitrification declined, resulting in linear N_2_ accumulation after approximately 40 h, the transcription of denitrification genes showed a subtle, and variable increase, particularly visible in *nirK*. Gene expression dropped to low levels (1E0–1E3 copies ng^–1^ RNA) after nitrate depletion (78 h).

### N_2_OR Activity During the Semi-Aerobic Phase

**FIGURE 5:**
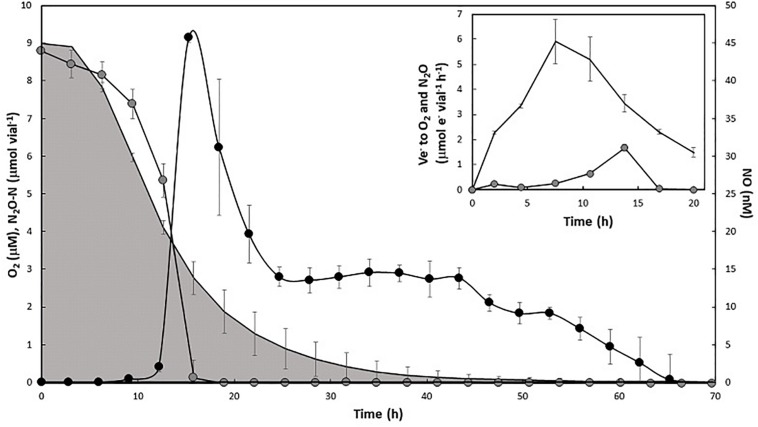
Aerobic N_2_O reduction in aerobically pre-cultured cells with 1% N_2_O and O_2_ injected in the headspace. Main panel: O_2_ concentration in liquid (μM, shaded area), N_2_O-N μmol vial^–1^ (gray circles), and NO nM in liquid (black circles). Standard deviation (*n* = 4) is shown as vertical lines. Insert: estimated rates of electron flow (Ve^–^, μmol e^–^ vial^–1^ h^–1^) to O_2_ (black line, no symbol) and N_2_O (gray circles) during the first 20 h of incubation.

The early expression of *nosZ* begs the question whether N2OR is active in the presence of μM concentrations of O_2_, preceding the “upstream” enzymes NIR and NOR. To test this, aerobically grown cells were transferred to vials injected with approximately 1 vol% each of O_2_ and N_2_O in headspace and containing 5 mM KNO_3_ in the medium. Due to differences in solubility, the initial concentrations of O_2_ and N_2_O in the liquid were approximately 9 and 20 μM, respectively. During the first 8 h after inoculation, the decrease in N_2_O was chiefly attributable to dilution by sampling. However, as O_2_ concentration reached approximately 50% of the initial concentration (4.13 μM in liquid), N_2_O was rapidly reduced and its exhaustion coincided with the appearance of NO (Figure 5). The O_2_ and N_2_O reduction rates were used to estimate the respective electron flow rates (Ve^–^, μmol e^–1^ vial^–1^ h^–1^) to O_2_ and N_2_O (Figure 5, insert). Initially, the electron flow was directed toward O_2_ as the only terminal acceptor. However, following the peak in Ve- to O_2_ (at approximately 9 h) Ve- to N_2_O increased and peaked while Ve- to O_2_ was still at approximately 50% of the observed maximum. Thus, there was parallel respiration of O_2_ and N_2_O for several hours, apparently preceding induction of NIR (NO increase), reflecting the observed early transcription of *nosZ*.

## Discussion

Denitrifying organisms are widespread and highly diverse. Even so, detailed studies of their physiology and biochemistry have chiefly been limited to a relatively small number of bacteria with mesophilic lifestyles. Thus, the knowledge of this pathway in extremophilic microorganisms, especially archaea, is scarce. The first studies on this topic in haloarchaea were reported in the late eighties, last century, using *Haloferax* species, and mainly *Haloferax denitrificans*, as microbe of study (Mancinelli and Hochstein, 1986; Tomlinson et al., 1986). At that time, it was shown that *H. denitrificans* was capable of anaerobic growth in the presence of nitrate (or nitrite) and that this was accompanied by the production of dinitrogen. In the presence of high concentrations of nitrate (i.e., 0.5%), nitrous oxide and nitrite were also detected (Tomlinson et al., 1986). During the last decade, denitrification in haloarchaea has gained new attention (Nájera-Fernández et al., 2012; Oren and Hallsworth, 2014; Torregrosa-Crespo et al., 2016). Recently, *H. mediterranei* was identified as a promising candidate model organism for haloarchaeal denitrification (Torregrosa-Crespo et al., 2019) and the present study elaborates on its respiratory physiology.

*Haloferax mediterranei* maintained aerobic growth in the presence of high nitrate (mM to M) and mM nitrite concentrations, although negatively affected by the latter (Figure 3A and Supplementary Figure S1). During the transition to denitrification and subsequent nitrate/nitrite respiration, it displayed a robust phenotype in terms of accumulation of N-oxide intermediates at near-neutral pH and with mM concentrations of N-oxyanions in the medium. Initial nitrate concentration had little effect on transient NO accumulation and no discernible effect on the NO concentration during the anoxic phase (Figure 1A), reflecting a well-orchestrated denitrification apparatus tuned to control the accumulation of the most toxic intermediate. In contrast, and not unexpectedly, high initial nitrite concentrations did have a clear positive effect on NO accumulation. Even so, NO toxicity apparently only came into play for the highest concentrations tested, in which case it led to complete arrest (10 and 40 mM NO_2_^–^ Supplementary Figure S4). Thus, *H. mediterranei* is an organism showing a tolerance to nitrite like many heterotrophic denitrifiers (Chen et al., 2008, 2009; Nájera-Fernández et al., 2012).

*Haloferax mediterranei* was robust when facing high N-oxyanion concentrations in near-neutral medium (pH = 7.3) but was sensitive to acidification. When pH decreased from 7.3 to 6.5 in cultures supplemented with nitrate, there was an apparent generalized inhibition of all the N-oxide reductases (Supplementary Table S2A). This contrasts with other denitrifiers, such as Paracoccus *denitrificans*, where sub-neutral pH affects the function of N_2_OR more than the rest of the denitrification enzymes (Bergaust et al., 2010). When nitrite was added to the medium, *H. mediterranei* could not grow at the lowest pH values (6.0 and 5.7). In these cultures, cells could not even consume O_2_ as final electron acceptor. This may have been due to the formation of toxic substances, a candidate being nitrous acid (HNO_2_) derived from the protonation of nitrite at low pH (Reddy et al., 1983; Almeida et al., 1995).

A striking phenotypic trait was the conspicuous and reproducible decline in apparent specific growth rate after 1.5–2 generations of anoxic growth, irrespective of remaining nitrate/nitrite in the medium. This likely reflected lack of *de novo* synthesis of denitrification enzymes and thus dilution of per cell concentrations of the N-oxide reductases during subsequent cell division. From the observed accumulation of nitrogenous gases, and a steady increase in nitrite during late anoxia (Figures 2, 4A), restraint of NIR synthesis appeared to marginally precede, and be more dramatic than, the decline in NAR synthesis. Moreover, the subsequent declining rate of e- flow toward NIR indicates net degradation of the existing enzyme pool. This effect was less dramatic in NAR, suggesting that the enzyme is less prone to degradation under these conditions. Lack of *de novo* synthesis would not necessarily result in any immediate decline in cell growth. This would depend on the margin by which the actual number of proteins per cell exceeds the critical number required to operate the metabolic machinery at normal rate. Cell/biomass yield per mole nitrate reduced to N_2_ was not significantly affected by denitrification rate (Supplementary Figure S5). However, in complex systems, failure to maintain *de novo* synthesis of denitrification enzymes would likely be a competitive disadvantage.

The observation that NAR outperforms NIR is reconcilable with previous work showing that the haloarchaeal NAR is a robust enzyme operating at high rates (Lledó et al., 2004; Martínez-Espinosa et al., 2007). However, the mechanisms underlying the apparent failure to maintain exponential growth (as seen by the activity of the enzyme pool) during denitrification (Figure 1B) are elusive. The transcription data (Figure 4) rule out any direct downregulation of N-oxide reductase genes, and the decline was clearly not caused by nitrate limitation because it occurred irrespective of residual nitrate (up to high mM range) in the medium. Moreover, it was unlikely to be due to lack of nutrients because (i) the experiments were conducted in complex medium and (ii) previous results show that in cultures with higher initial oxygen (7 vol%) and thus higher cell density, 5 mM nitrate was reduced to N_2_ without any premature decline in growth rate (Supplementary Figure S6). However, in contrast to cultures with 1% initial O_2_, cultures with 7% O_2_ depleted the available nitrate while conditions remained micro-oxic. Inspection of gas data from cultures with 1% initial oxygen shows that the decline in anaerobic growth rate typically co-occurs with oxygen exhaustion ([O_2_] < 0.1 μM in liquid). However, if micro-oxic conditions are required for *de novo* synthesis of N-oxide reductases, the critical threshold is likely considerably higher than 0.1 μM O_2_ because any observable effect would be delayed. The apparent O_2_ threshold for induction of denitrification increases with initial concentration and has been observed to be > 20 μM in cultures with 7% initial oxygen. In such cultures, O_2_ still remained at concentrations of approximately 5 μM in the liquid at depletion of N-oxides (Supplementary Figure S6). Put together, the data suggest that *H. mediterranei* requires the presence of low μM concentrations of oxygen for *de novo* synthesis of denitrification enzymes (NAR and NIR), and that this regulation/effect is post-transcriptional. In fact, we did observe an increase, albeit subtle, in e- flow to N-oxides after O_2_ injection to anaerobic cultures (Supplementary Figure S2).

**FIGURE 6:**
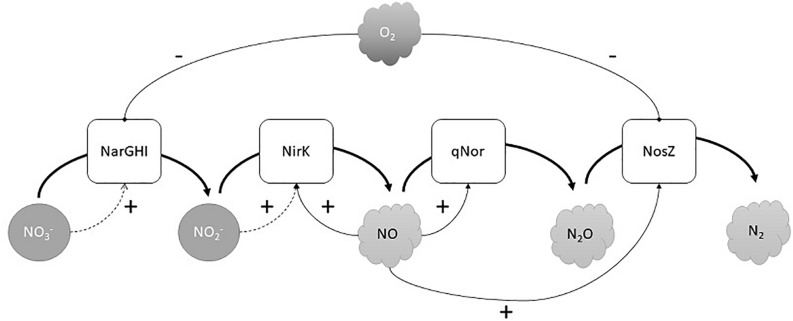
Tentative regulatory model of denitrification in *H. mediterranei*. (+) means a positive regulation; (-) means negative regulation.

To begin to understand the transcriptional regulation of denitrification in *H. mediterranei*, we quantified the expression of *narG*, *nirK*, *norZ*, and *nosZ* during the oxic-anoxic transition and subsequent anaerobic growth. Sampling was guided by observed gas kinetics, yielding a high-resolution profile of gene expression vs denitrification activity. Both *narG* and *nosZ* appeared to be induced directly by hypoxia and did not require the presence of NO or nitrite for expression (Figure 4). Thus, *narG* and *nosZ* are likely activated via an oxygen (and possibly nitrate) sensor. The negative regulation of NAR by O_2_ via O_2_-sensing transcription factors is common in denitrifying bacteria (Spiro, 2012) and has also been observed for N_2_OR (Bergaust et al., 2010, 2012). In *H. mediterranei*, the AcrR-like transcriptional regulator occupies the same position as the NarO regulator in *Haloferax volcanii*, recently identified as a putative O_2_ sensor in this strain (Hattori et al., 2016). The “core” reductases, NIR and NOR, were not induced by hypoxia alone, but apparently required the presence of NO (and possibly nitrite). The appearance of NO also coincided with a second peak in *nosZ* transcription. Thus, *nirK*, *norZ*, and *nosZ* all appear to be under the control of unknown NO/nitrite sensor(s), again resembling bacterial denitrifiers in their regulatory circuits (van Spanning et al., 1999; Zumft, 2002). Positive *nirK* regulation by nitrite may underlie the second peak in *nirK* expression during late anoxia when nitrite accumulates (Figure 4). Due to the sequential nature of denitrification, it seems intuitive that the synthesis of N-oxide reductases follows the same order. This has been shown to not always be the case in bacteria, where N2OR activity may precede NIR and NOR during the transition to anoxia (Qu et al., 2016). *H. mediterranei* displayed a similar phenotype where active N_2_OR was produced during the semi-aerobic phase, independent of NO/nitrite induction (Figure 5). The transcriptional regulation of denitrification in *H. mediterranei* is tentatively summarized in Figure 6.

*Haloferax mediterranei* emerges as a suitable model organism for the study of denitrification in saline and hypersaline environments. It tolerates the presence of high concentrations of nitrates and nitrites at optimum pH but appears to require micro-oxic conditions to sustain anaerobic growth by denitrification. It seems, as previous studies point out (Chen and Strous, 2013), that denitrification is not an entirely anaerobic process in all organisms. In some organisms such as *H. mediterranei*, instead of providing an exclusive and separate alternative to oxygen respiration, it may be a parallel process under micro-oxic conditions as a means to derive additional energy, but ineffective under strict anoxia. More studies will be needed to confirm the hypothesized requirement for oxygen for *de novo* synthesis of N-reductases. *H. mediterranei* appears to have many regulatory traits in common with its bacterial counterparts, but in-depth molecular, physiological, and biochemical studies are still needed to understand the respiratory metabolism of this extremophile.

## Data Availability Statement

All datasets generated for this study are included in the article/Supplementary Material.

## Author Contributions

RM-E and LB conceived the experiments, were the project administrators, and oversaw the funding acquisition. The design of the experiments was conducted by LB and JT-C. The experiments were done by JT-C. Results were analyzed by all the authors. All the authors contributed to the discussion of the results and manuscript writing.

## Conflict of Interest

The authors declare that the research was conducted in the absence of any commercial or financial relationships that could be construed as a potential conflict of interest.
