# Supplementary material for: Haloferax mediterranei, an Archaeal Model for Denitrification in Saline Systems, Characterized Through Integrated Physiological and Transcriptional Analyses
Source: Front Microbiol. 2020 Apr 22;11:768. doi: 10.3389/fmicb.2020.00768 (PMC7188791; doi:10.3389/fmicb.2020.00768)
Supplement: Supplementary file 1 [file Data_Sheet_1.docx]

Supplementary Material

**Supplementary Table 1.** A) Phenotypic parameters in *Haloferax mediterranei* cultures monitored during the transition from aerobic growth (initial O_2_: 1% in headspace) to denitrification in nitrate-supplemented media (from 2 to 2000 mM KNO_3_). B) Apparent specific aerobic and anaerobic growth rates (µ_ox_*_,_* µ_anox_ – h^-1^)*,* generation times (G_ox_, G_anox_ – h^-1^*)* and number of generations of anoxic growth during log linear growth.

**(A)**

| **Initial NO_3_^-^**  **(mM)** | **NO_2_^-^ _max_ (μM)** | | **N_2_O _max_ (nmol vial^-1^)** | | **NO _max_ (nM)** | | **NO _steady_ (nM)** | |
| --- | --- | --- | --- | --- | --- | --- | --- | --- |
|  | Average | St. Dev. | Average | St. Dev. | Average | St. Dev. | Average | St. Dev. |
| **2** | 38 | 21 | 107 | 54 | 65 | 12 | 17 | 2 |
| **5** | 535 | 154 | 41 | 32 | 52 | 14 | 16 | 2 |
| **10** | 2585 | 275 | 57 | 17 | 45 | 11 | 16 | 2 |
| **20** | 2081 | 1967 | 133 | 70 | 66 | 19 | 19 | 2 |
| **200** | 5399 | 2652 | 214 | 31 | 105 | 16 | 21 | 2 |
| **2000** | 257 | 161 | 1062 | 76 | 105 | 7 | 24 | 2 |

**(B)**

| **Initial NO_3_^-^**  **(mM)** | **µ_ox_ (h^-1^)** | | **µ_anox_ (h^-1^)** | | **G(t)_ox_ (h)** | | **G(t)_anox_ (h)** | | **Generations of anoxic growth** | |
| --- | --- | --- | --- | --- | --- | --- | --- | --- | --- | --- |
|  | Average | St. Dev. | Average | St. Dev. | Average | St. Dev. | Average | St. Dev. | Average | St. Dev. |
| **2** | 0.207 | 0.024 | 0.039 | 0.013 | 3.383 | 0.411 | 19.835 | 7.756 | 1.571 | 0.270 |
| **5** | 0.209 | 0.011 | 0.046 | 0.007 | 3.317 | 0.173 | 15.262 | 2.352 | 1.817 | 0.229 |
| **10** | 0.207 | 0.022 | 0.044 | 0.002 | 3.373 | 0.360 | 15.687 | 0.681 | 1.847 | 0.127 |
| **20** | 0.198 | 0.019 | 0.037 | 0.017 | 3.532 | 0.268 | 19.438 | 3.857 | 1.602 | 0.215 |
| **200** | 0.178 | 0.009 | 0.032 | 0.006 | 3.892 | 0.183 | 22.316 | 3.994 | 1.802 | 0.150 |
| **2000** | 0.115 | 0.019 | 0.011 | 0.003 | 6.125 | 1.003 | 64.236 | 14.903 | 1.480 | 0.348 |

**Supplementary Table 2.** A) Phenotypic parameters in *H. mediterranei* cultures monitored during the transition from aerobic growth (initial O_2_: 1% in headspace) to denitrification in nitrate supplemented media (2mM KNO_3_) with different pH values (7.3; 7.0; 6.5; 6.0; 5.7). The buffer used was Bis-Tris 100 mM. Values are presented as average of cultures by triplicate (*n=3*) with standard deviation. B) Phenotypic parameters in *H. mediterranei* cultures monitored during the transition from aerobic growth (initial O_2_: 1% in headspace) to denitrification in nitrite supplemented media (2mM KNO_2_) with different pH values (7.3; 7.0; 6.5; 6.0; 5.7). The buffer used was Bis-Tris 100 mM. Values are presented as average of cultures by triplicate (*n=3*) with standard deviation. *n.a. not applicable.*

**(A)**

|  | **pH** | | | | | | | | | |
| --- | --- | --- | --- | --- | --- | --- | --- | --- | --- | --- |
|  | **7.3** | | **7.0** | | **6.5** | | **6.0** | | **5.7** | |
|  | Average | St. Dev. | Average | St. Dev. | Average | St. Dev. | Average | St. Dev. | Average | St. Dev. |
| O_2init_  (μM liquid) | 1.58 | 0.14 | 2.18 | 0.29 | 2.16 | 0.73 | 3.29 | 1.00 | 2.63 | 0.89 |
| NO_2_^-^_max_  (μmol vial^-1^) | 77.05 | 5.09 | 31.91 | 2.26 | 5.67 | 3.23 | 15.07 | 1.58 | 9.03 | 1.86 |
| NO_max_  (nM in liquid) | 32.87 | 4.83 | 24.22 | 2.94 | 15.37 | 4.14 | 105.60 | 4.70 | 105.21 | 14.86 |
| N_2_O_max_  (nM in liquid) | 307.43 | 33.77 | 148.49 | 54.06 | 142.15 | 42.54 | 0.22 | 0.17 | 0.61 | 0.25 |
| % initial NO_3_^-^ reduced to N_2_ | 100.00 | 0.00 | 100.00 | 0.00 | 37.60 | 24.08 | 92.65 | 3.59 | 51.24 | 16.56 |

**(B)**

|  | **pH** | | | | | | | | | |
| --- | --- | --- | --- | --- | --- | --- | --- | --- | --- | --- |
|  | **7.3** | | **7.0** | | **6.5** | | **6.0** | | **5.7** | |
|  | Average | St. Dev. | Average | St. Dev. | Average | St. Dev. | Average | St. Dev. | Average | St. Dev. |
| O_2init_  (μM liquid) | 1.40 | 0.34 | 2.58 | 0.51 | 2.76 | 0.05 | n.a. | | | |
| NO_max_  (nM in liquid) | 489.76 | 37.76 | 769.71 | 198.56 | 960.38 | 141.96 | 254.72 | 53.36 | 367.43 | 5.05 |
| N_2_O_max_  (nM in liquid) | 220.57 | 17.45 | 149.36 | 23.95 | 228.27 | 80.34 | 200.53 | 63.09 | 281.52 | 2.47 |
| % initial NO_2_^-^ reduced to N_2_ | 100.00 | 00.00 | 100.00 | 00.00 | 80.47 | 33.82 | 2.73 | 3.77 | 2.10 | 2.59 |

**
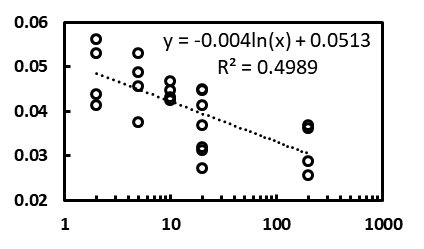

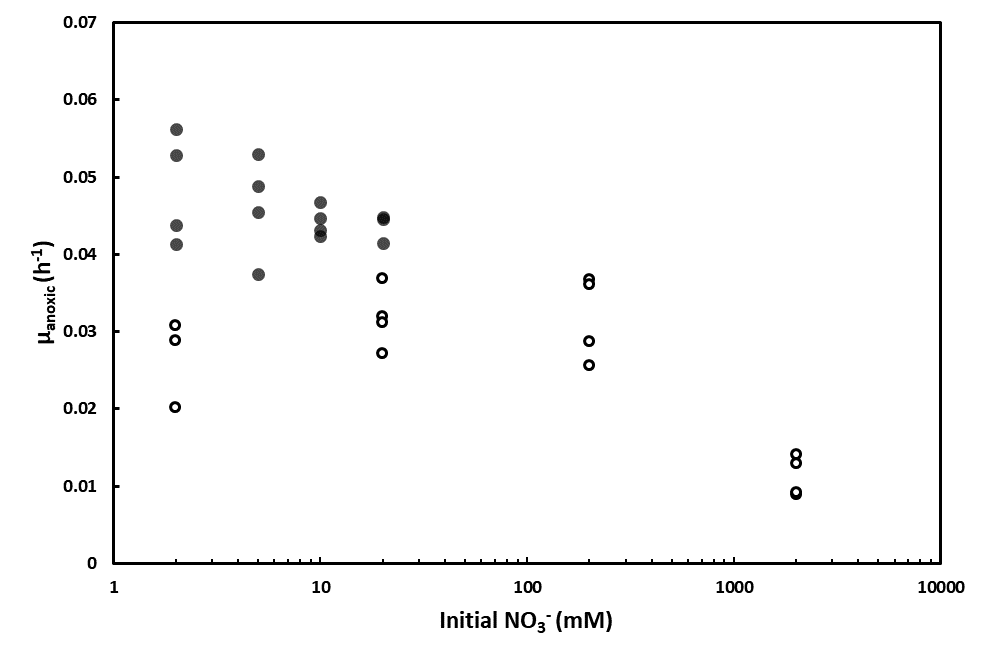
**

**Supplementary Figure 1.** Apparent anaerobic growth rate by denitrification (µ_anox_) in H. mediterranei exposed to KNO_3_ concentrations ranging from 2 to 2000 mM. Each circle represents one culture. The main panel summarized data from two separate experiments, open and closed symbols, respectively. The insert shows the weak negative trend seen when disregarding the three deviant 2 mM cultures (open circles, main panel). µ_anox_ was estimated based on the exponential slope of the total e- flow to terminal electron acceptors during denitrification.


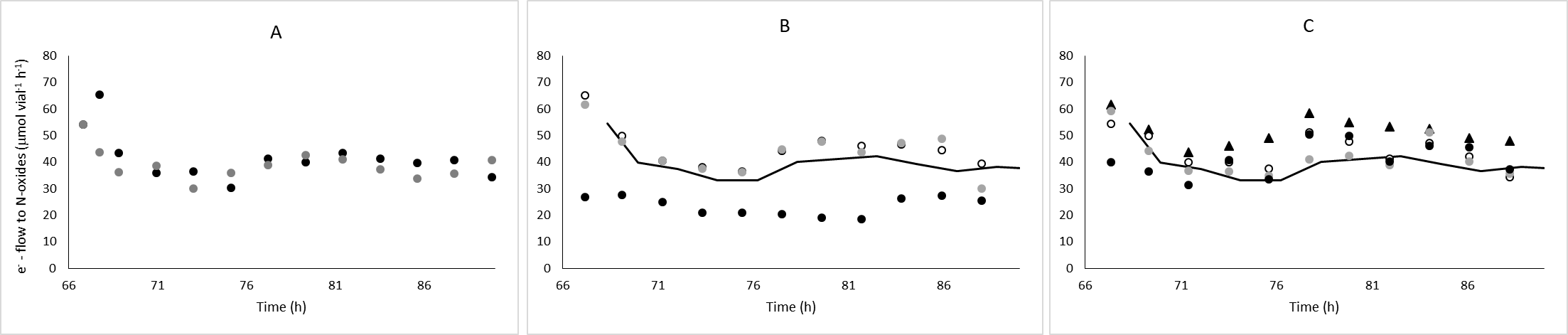


**Supplementary Figure 2.** e^-^ - flow to terminal N-oxides (μmol vial^-1^ h^-1^) during denitrification in nitrate-supplemented media (5 mM KNO_3_) and 1 vol% initial O_2_. Panel A: e^-^ - flow to terminal N-oxides of two different vials (black and grey circles); Panel B: e^-^ - flow to terminal N-oxides of three different vials (black, grey and open black circles) in which NO (concentration) was injected in the headspace at 68 h of incubation (line represents the average of e^-^ - flow of the two vials of panel A, of which headspace remained untouched); Panel C: e^-^ - flow to terminal N-oxides of four different vials (black, grey, open black circles and black triangles) in which O_2_ (concentration) was injected in the headspace at 68 h of incubation (line represents the average of e^-^ - flow of the two vials of panel A, of which headspace remained untouched).


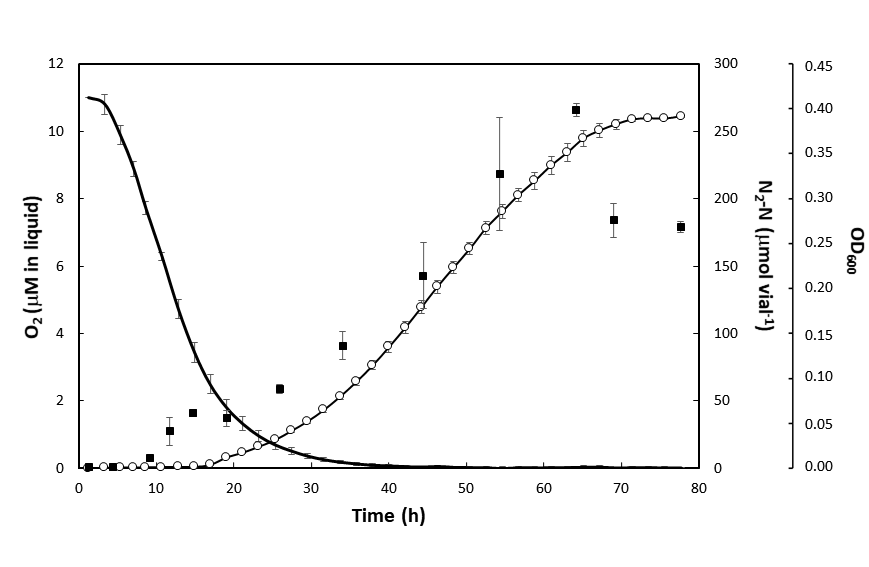
**(A)**

**Supplementary Figure 3.** Gas kinetics to terminal e-acceptors during the transition to anoxia in media with 1 vol% initial O_2_ supplemented with 5mM KNO_3_ (*n=3*) (A) and 2mM KNO_2_ (*n=3*) (B): consumption of O_2_ (black line, no symbols), production of N_2_ (open circles) and OD_600_ measurements (black squares).

**Supplementary Figure 3.** Gas kinetics to terminal e-acceptors during the transition to anoxia in media with 1 vol% initial O_2_ supplemented with 5mM KNO_3_ (*n=3*) (A) and 2mM KNO_2_ (*n=3*) (B): consumption of O_2_ (b line, no symbols), production of N_2_ (open circles) and OD_600_ measurements (black squares).

**(B)**


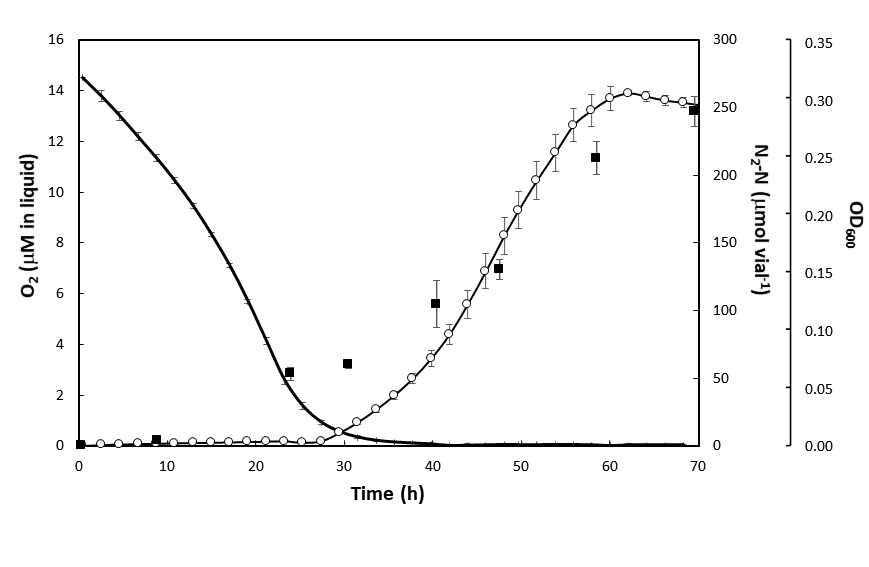


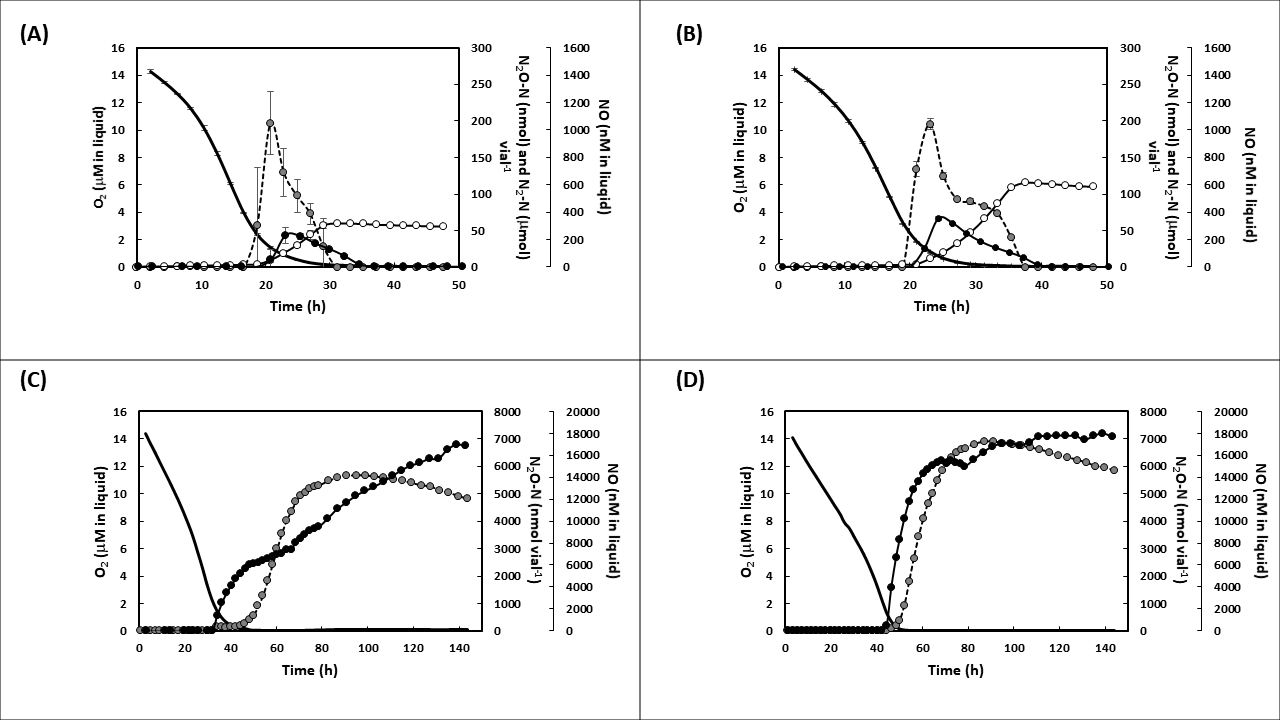
**Supplementary Figure 4.** Gas kinetics to terminal e-acceptors during the transition to anoxia in media with 1 vol% initial O_2_ and different initial nitrite concentrations: (A) (1 mM KNO_2_), (B) (2 mM KNO_2_), (C) (10 mM KNO_2_), (D) (40 mM KNO_2_). Consumption of O_2_ (black line, no symbols) and subsequent accumulation of N-oxides (NO -closed black circles; N_2_O –closed grey circles; N_2_ – open circles). The experiment was conducted at 35^o^C and in triplicate batch cultures. In panels C and D, N_2_ accumulation is not shown because it was near zero: one representative replicate is displayed.

**
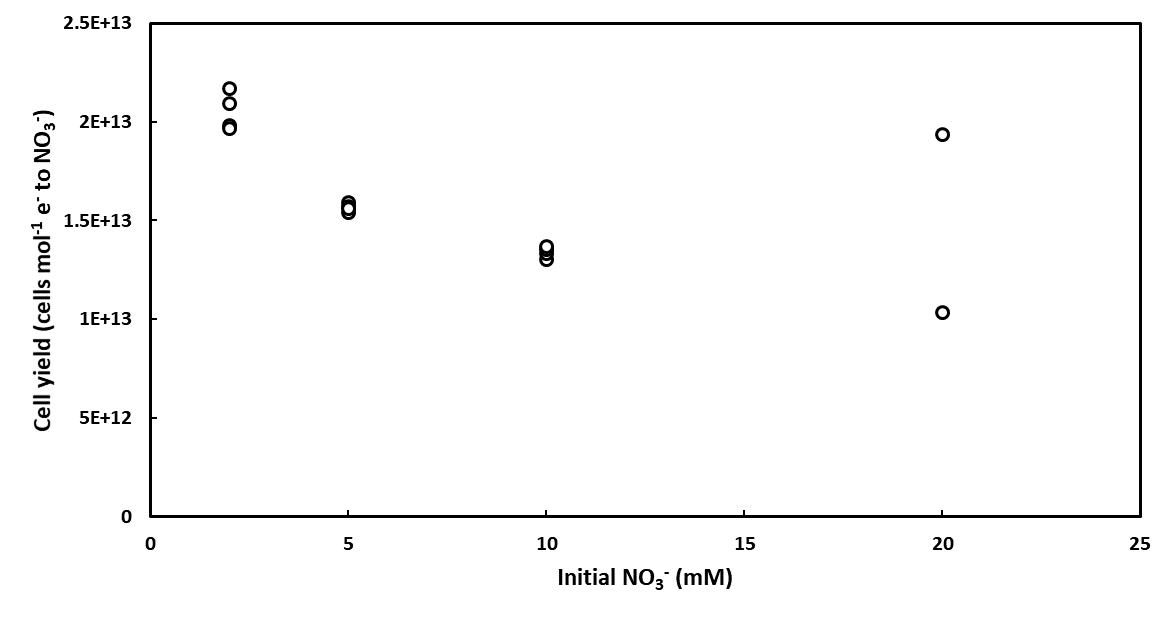
**

**Supplementary Figure 5.** Cell yield (cells mol^-1^ e^-1^ to NO_x_) of *H. mediterranei* exposed to KNO_3_ initial concentrations ranging from 2 to 2000 mM. Each circle represents one culture.

**Cell yield (cells mol^-1^ e^-^ to NO_x_)**

**
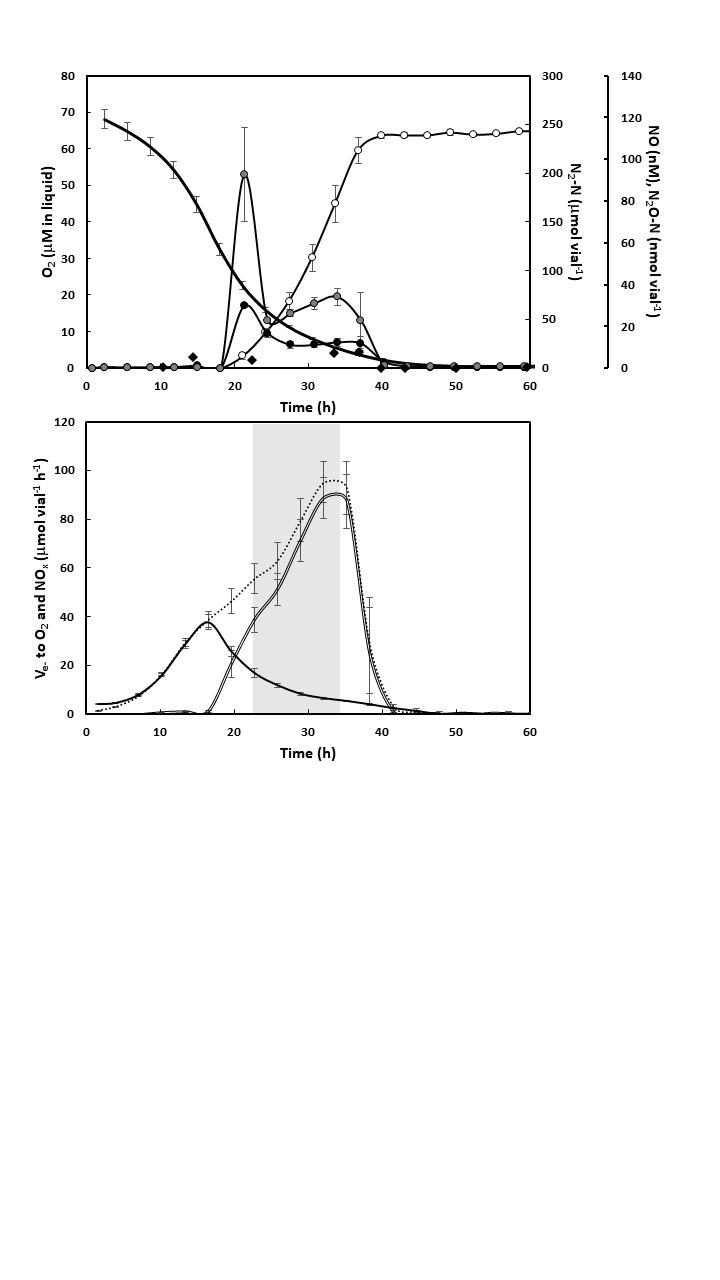
**

**Supplementary Figure 6.** Gas kinetics and e-flow to terminal e-acceptors during the transition to anoxia in nitrate-supplemented medium. Top panel: consumption of O_2_ (black line, no symbols) and accumulation of N-oxides (NO- closed black circles; N_2_O- closed grey circles; N_2_- open circles) with 7% initial O_2_ in headspace and 5mM KNO_3_ in the medium. The experiment was conducted at 35^o^C and in triplicate batch cultures. Bottom panel shows e-flow (μmol vial^-1^ h^-1^) to O_2_ (black line), N-oxides (double black line) and the total e-flow (discontinuous black line). The apparent exponential growth by denitrification is shown as a grey area (22-33 h).

**Supplementary Table 3.** List of primers used for gene expression analysis.

| Gene | Primer sequences (5’→3’) | |
| --- | --- | --- |
| *narG* | Forward: | ACTACTTCAACCAAGCCAAAGG |
|  | Reverse: | ATTTGCCAGTCGGTCTTCG |
| *nirK* | Forward: | TCGCTGAGTACGGACCCA |
|  | Reverse: | TACAACCCTTGCGTGTGACA |
| *norZ* | Forward: | TTGCGAAGACCTGGCATATC |
|  | Reverse: | CGAGGGTGACGACGACGAT |
| *nosZ* | Forward: | CGAAGTGGACGGCAACCTC |
|  | Reverse: | TTGTCGCCCTTGTTGAGTGA |
